# The telomere-to-telomere gap-free reference genome of wild blueberry (*Vaccinium duclouxii*) provides its high soluble sugar and anthocyanin accumulation

**DOI:** 10.1093/hr/uhad209

**Published:** 2023-10-10

**Authors:** Tuo Zeng, Zhijiao He, Jiefang He, Wei Lv, Shixiang Huang, Jiawen Li, Liyong Zhu, Shuang Wan, Wanfei Zhou, Zhengsong Yang, Yatao Zhang, Chong Luo, Jiawei He, Caiyun Wang, Liangsheng Wang

**Affiliations:** School of Life Sciences, Guizhou Normal University, Guiyang 550000, China; Institute of Alpine Economic Plant, Yunnan Academy of Agricultural Sciences, Lijiang 674199, Yunnan, China; School of Life Sciences, Guizhou Normal University, Guiyang 550000, China; School of Life Sciences, Guizhou Normal University, Guiyang 550000, China; School of Life Sciences, Guizhou Normal University, Guiyang 550000, China; School of Advanced Agricultural Sciences, Peking University, 100871 Beijing, China; National Key Laboratory for Germplasm Innovation & Utilization of Horticultural Crops, College of Horticulture & Forestry Sciences, Huazhong Agricultural University, Wuhan 430070, China; Wuhan Benagen Technology Co., Ltd, Wuhan 430070, China; National Key Laboratory for Germplasm Innovation & Utilization of Horticultural Crops, College of Horticulture & Forestry Sciences, Huazhong Agricultural University, Wuhan 430070, China; Institute of Alpine Economic Plant, Yunnan Academy of Agricultural Sciences, Lijiang 674199, Yunnan, China; School of Life Sciences, Guizhou Normal University, Guiyang 550000, China; School of Life Sciences, Guizhou Normal University, Guiyang 550000, China; Institute of Alpine Economic Plant, Yunnan Academy of Agricultural Sciences, Lijiang 674199, Yunnan, China; National Key Laboratory for Germplasm Innovation & Utilization of Horticultural Crops, College of Horticulture & Forestry Sciences, Huazhong Agricultural University, Wuhan 430070, China; Key Laboratory of Plant Resources, Institute of Botany, Chinese Academy of Sciences, Beijing 100093, China; China National Botanical Garden, Beijing 100093, China; University of Chinese Academy of Sciences, Beijing 100049, China

## Abstract

*Vaccinium duclouxii*, endemic to southwestern China, is a berry-producing shrub or small tree belonging to the Ericaceae family, with high nutritive, medicinal, and ornamental value, abundant germplasm resources, and good edible properties. In addition, *V. duclouxii* exhibits strong tolerance to adverse environmental conditions, making it a promising candidate for research and offering wide-ranging possibilities for utilization. However, the lack of *V. duclouxii* genome sequence has hampered its development and utilization. Here, a high-quality telomere-to-telomere genome sequence of *V. duclouxii* was *de novo* assembled and annotated. All of 12 chromosomes were assembled into gap-free single contigs, providing the highest integrity and quality assembly reported so far for blueberry. The *V. duclouxii* genome is 573.67 Mb, which encodes 41 953 protein-coding genes. Combining transcriptomics and metabolomics analyses, we have uncovered the molecular mechanisms involved in sugar and acid accumulation and anthocyanin biosynthesis in *V. duclouxii*. This provides essential molecular information for further research on the quality of *V. duclouxii*. Moreover, the high-quality telomere-to-telomere assembly of the *V. duclouxii* genome will provide insights into the genomic evolution of *Vaccinium* and support advancements in blueberry genetics and molecular breeding.

## Introduction

Blueberry (*Vaccinium* spp.), belonging to the Ericaceae family, is a small, perennial fruit tree with a delicate pulp, sweet and sour taste, a rich nutrient profile, high levels of calcium, iron, vitamins, and other trace elements, providing numerous health benefits such as liver protection, anticancer properties, antioxidant effects, and anti-aging properties [1]. Aside from its nutritional and medicinal value, blueberry also possesses high ornamental value [2]. China is the largest producer of blueberry in the world, with a cultivated area of 66 400 hectares in 2020, accounting for 29.24% of the global cultivated area, and a total annual output of 347 200 tons [3]. Therefore, blueberries have become a horticultural crop of significant industrial value with immense development potential.

The cultivated blueberry gene pool is mainly composed of common cultivated varieties, such as *Vaccinium corymbosum* (2*n* = 4*x* = 48; northern highbush blueberry), *V. angustifolium* (2*n* = 4*x* = 48), and *V. virgatum* (2*n* = 4*x* = 48) [4]. The limited diversity within this gene pool poses various constraints on blueberry breeding efforts. However, wild blueberry varieties exhibit genetic diversity regarding disease resistance, adaptability, yield, and flavor. Therefore, leveraging the genetic resources of wild blueberries for breeding holds significant importance in enhancing the stress resistance and quality of cultivated varieties.

The genus *Vaccinium* encompasses a total of 779 accepted species (https://wfoplantlist.org/) and exhibits a wide distribution across the world. Certain wild *Vaccinium* species have been used in blueberry breeding, such as *V. darrowii* in developing southern highbush blueberries [4]. Among these species, *V. bracteatum* (2*n* = 2*x* = 24), a native diploid species originating from East Asia, has characteristics such as pigmented fruit pulp with high antioxidant capacity and tolerance to high-pH soils [4]. Tetraploid derivatives of *V. bracteatum* can hybridize with *V. corymbosum*, resulting in hybrids with higher concentrations of soluble solids than the cultivated parent ‘Spartan’ [5]. These hybrids also exhibit enhanced pH tolerance [6] and anthocyanin content within the fruit pulp [7]. These attributes highlight the significance of wild blueberries in breeding endeavors.

**Figure 1:**
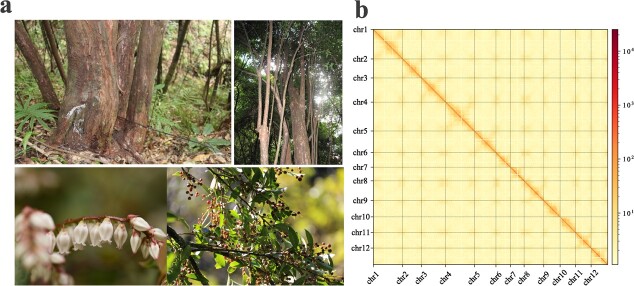
Overview of *V. duclouxii* trees and genome. **a** Photographs of ancient tall *V. duclouxii* trees in the Yunnan Province of China (27°15′23.08″ N, 99°35′44.73″ E, altitude 2081 m; upper panel), and close-up pictures showing the morphology of flowers, leaves, and berries (lower panel). **b** Hi-C heat map of chromosome interactions. The x and y axes represent the ordered positions of all 12 chromosomes.

*Vaccinium duclouxii* (2*n* = 2*x* = 24) is a wild blueberry native to southwestern China, primarily found in Yunnan, and Sichuan provinces, and is a dominant species in the Ailao Mountains of Yunnan [8]. It thrives in yellow clay soil with a thin layer, low fertility, and poor permeability, demonstrating exceptional environmental adaptability and remarkable stress resistance [9]. Despite competition from local tree species and challenging environmental conditions, it demonstrates remarkable stress resistance [9]. These plants grow as shrubs or small trees on hillsides within evergreen broadleaved forests at an altitude ranging from 1550 to 2600 m [10], with typical heights reaching 1–5 m [11]. In Yunnan Province, ancient trees taller than 10 m can be found (Fig. 1a). One notable characteristic of *V. duclouxii* berries is their higher sulfur and anthocyanin content than commonly cultivated blueberries [12]. The total sugar content of *V. duclouxii* is ~13.8%, significantly higher than *V. fragile* (7.38%) and *V. ashei* (9.24%). Additionally, its total acid content of 4% surpasses that of *V. fragile* (1.72%) and *V. ashei* (0.58%) [10]. Like *V. bracteatum*, *V. duclouxii* fruit pulp is enriched with pigments that provide exceptional antioxidant capabilities. Moreover, the induced tetraploid *V. duclouxii* exhibits distinct advantages over its diploid predecessor, including thicker stems and longer leaves [9]. These characteristics indicate its tremendous breeding value. However, the lack of a genome has significantly hindered further study on this species.

Recent genomic studies revealed the complete genomes of several *Vaccinium* species, including *V. darrowii* (2*n* = 2*x* = 24; major parent of the southern highbush blueberry cultivar) [13, 14], *V. corymbosum* [15], *V. macrocarpon* (2*n* = 2*x* = 24) [16], and *V. myrtillus* (2*n* = 2*x* = 24) [17]. However, studies on wild blueberries native to China are limited, with only *V. bracteatum* (2*n* = 2*x* = 24) having its genome sequence identified [18]. Notably, a precise telomere-to-telomere (T2T) genome of blueberry is currently lacking.

The advancement of ultra-long read sequencing technology has revolutionized genome assembly, allowing the generation of complete, gap-free T2T assemblies in various plant species, including *Arabidopsis thaliana* [19], rice [20], watermelon [21], kiwifruit [22], banana [23], bitter melon [24], strawberry [25], and *Rhodomyrtus tomentosa* [26]. T2T assembly refers to constructing high-quality genomes that capture all centromeres and repetitive regions with exceptional accuracy, continuity, and integrity [27]. T2T assemblies and their precise reconstruction of repetitive regions offer a comprehensive understanding of centromeres and telomeres, enable annotation of a large number of protein-coding genes, facilitate comparative genomics and evolutionary biology, and provide precise genome sequences for genetic domestication and breeding.

In this study, we utilized the latest sequencing technologies, including ultra-long Oxford Nanopore Technology (ONT), Pacific Bioscience (PacBio), and chromosome conformation capture (3C)-based (Hi-C) sequencing, to generate a high-quality, contiguous, T2T genome assembly of *V. duclouxii*. This remarkable feat has allowed us to explore the telomeric and centromeric regions of *V. duclouxii*. Beyond the scientific implications, our research on *V. duclouxii* has significant industrial relevance, particularly in advancing the cultivation and genetic improvement of high-quality blueberry varieties. Our work lays a solid foundation for future investigation of this vital crop species.

## Results

### Genome assembly and quality assessment

*Vaccinium duclouxii* (voucher specimen SGLD20220023 was deposited in the Kunming Institute of Botany), planted in the nursery of Yunnan Mountain Institute, was selected for T2T gap-free genome assembly (Fig. 1a). A genome survey indicated that the genome of *V. duclouxii* is ~571.62 Mb, with 1.55% heterozygosity (Supplementary Data Fig. S1). To obtain a highly contiguous genome assembly of *V. duclouxii*, we leveraged multiple sequencing platforms and combined the data. Specifically, we generated a total of 27.19 Gb (~44.65×) PacBio High Fidelity (HiFi) reads, 41.57 Gb (~61.32×) ONT ultra-long reads, and 24.49 Gb targeted ONT reads using the PacBio Sequel II and ONT platforms, respectively. The N50 length of HiFi reads was >20.72 kb, while the N50 length of ONT reads was as high as 49.168 kb. Additionally, we constructed a Hi-C library using pairing terminal technology, resulting in a high-quality dataset of 65.56 Gb (~114.29×) reads for downstream scaffolding, validation, and orientation of contigs (Supplementary Data Table S1). The heat map of the Hi-C assembly showed that the genome assembly is complete (Fig. 1b). Overall, a 573.67 Mb gap-free genome of *V. duclouxii* was generated, representing all 12 chromosomes, with a contig N50 length of 48.51 Mb (Table 1).

**Table 1:** Summary statistics of *V. duclouxii* genome assemblies.

**Genomic feature**	**Value**
Total size of assembled contigs (Mb)	583.19
Number of contigs	63
N50 value of contig length (Mb)	22.19
Total size of assembled genomes (Mb)	573.67
Number of base chromosomes	12
Number of gap-free chromosomes	12
Number of candidate telomeres	23
Number of candidate centromeres	12
TE size (%)	55.27
GC content (%)	38.75
Genome BUSCOs (%)	98.50
Number of genes	41 953
Gene BUSCOs (%)	98.10

**Figure 2:**
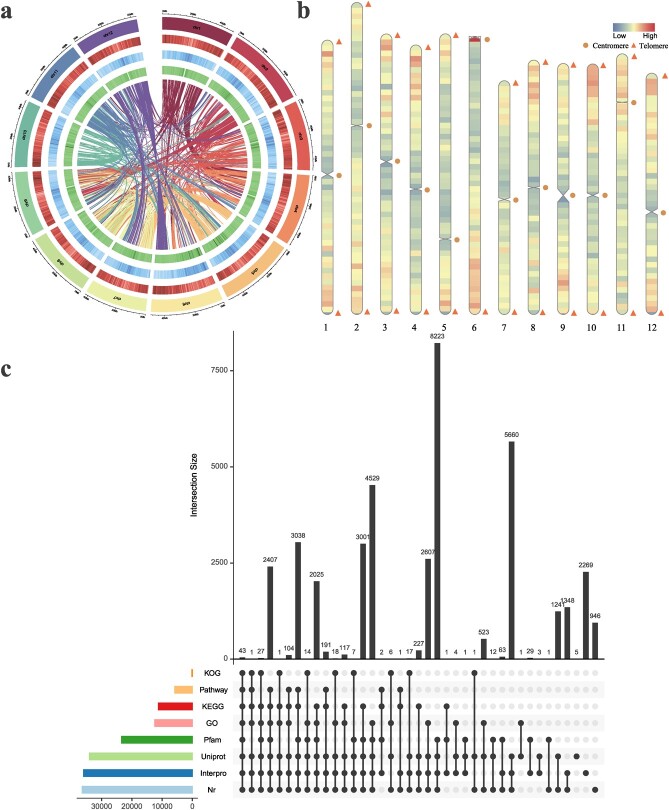
High-quality T2T genome assembly of *V. duclouxii*. **a** Chromosome ideogram. Concentric circles represent chromosome, gene density, repeat content, and GC content from the outside to the inside. Lines in the interior indicate collinearity. **b** Telomere detection map. Triangles and circles represent telomeres and centromere within the assembled chromosomes; warm tones indicate regions of high gene density, while cool tones indicate regions of low gene density. **c** Upset plots of gene function annotation, gene number annotation in KOG, pathway, KEGG, GO, Pfam, UniProt, InterPro, and Nr.

The results of gene density, transposable element (TE) content, and GC content analyses are shown in Fig. 2a. The *V. duclouxii* genome size determined in this study using multiple sequencing platforms was slightly smaller than the flow cytometry-based estimate (~600 Mb) (Supplementary Data Fig. S1), which may be due to bias in estimating the size of small genomes. We utilized the tandem repeat sequencer v4.07b to identify candidate centromeric sequences from the assembled genome and confirmed that centromeric DNA was the most abundant tandem repeat. Using a seven-base telomeric repeat sequence as a query, we identified 23 telomeres (11 pairs, 1 single, Chr6 was an acrocentric chromosome). We also predicted 12 alternative centromeric tandem repeat sequences and present the repetitive sequences and telomeric regions in the supplementary material (Supplementary Data Tables S2 and S3). All of the chromosomes were assembled as gap-free chromosomes (Fig. 2b). Thus, the *V. duclouxii* genome could be considered a high-quality T2T assembly.

Multiple data types and methods were implemented to evaluate the quality of the *V. duclouxii* genome assembly. The interaction matrix generated from the Hi-C short-read library indicated that all 12 chromosomes were fully and reasonably assembled. Illumina short reads and HiFi reads showed a mapping rate of 99.42 and 99.98%, respectively. The results of BUSCO evaluation showed that the percentage of complete BUSCOs was 98.5 (Supplementary Data Table S4), and the assembly consensus quality value (QV) was 50.56 (Supplementary Data Table S5), indicating that the *V. duclouxii* genome assembly was of high integrity and accuracy. The completeness test of long terminal repeats (LTRs) showed that the LTR assembly index (LAI) value of the assembly was 20.22, which was higher than the LAI values of *Actinidia chinensis* and *R. tomentosa* (16.38 and 16.16, respectively) [22, 26]. This high-quality gapless genome could be used to elucidate the mysteries of these unclear regions.

### Genome annotation

Repetitive sequences, pseudogenes, non-coding RNAs, and homologous protein-coding genes and their functions were annotated using non-redundant (NR) protein sequence, evolutionary gene genealogy: Non-supervised Orthologous Groups (eggNOG), Gene Ontology (GO), Kyoto Encyclopedia of Genes and Genomes (KEGG), SwissProt, Pfam, and TrEMBL databases (Fig. 2c). Overall, 54.8% of the sequences that make up the *V. duclouxii* genome were predicted to be repetitive, most of which were tandem repeats, interspersed repeats, and TEs. Based on the constructed duplicate database, ~317 Mb represented TEs, and 178 Mb represented full-length LTRs, including LTR-Gypsy (13.74%), LTR-Copia (6.76%), and other sequences (Supplementary Data Table S6).

Homology-based prediction, transcriptome-based prediction, and *de novo* prediction, together, revealed a total of 41 953 genes, the average coding sequence (CDS) length was 1081.50 bp, and the average number of exons per CDS was 4.94 (Supplementary Data Table S7). Gene length frequency alignments with closely related species showed that our annotation results are reliable (Supplementary Data Fig. S2). A total of 92.28% of the obtained genes could be annotated from the database (Supplementary Data Table S8). Most gene annotations based on transcriptome and genome homology predictions indicated a high degree of confidence. A total of 456 tRNAs, 1136 rRNAs, and 222 miRNAs were identified (Supplementary Data Table S9).

### Comparative genome analysis

Identification of homologous genes is an important aspect of evolutionary analysis. By identifying homologous genes and cluster analysis of gene families, we can obtain single-copy and multiple-copy gene families, which are relatively conserved among species, and species-specific gene families. We identified 71 830 orthologous gene families, comprising 494 375 genes, in all species. Among these, 6601 gene families, comprising 183 572 genes, were common to all species, while 3188 gene families, comprising 5060 genes, were specific to *V. duclouxii* (Supplementary Data Table S10).

To gain insight into the functional roles of *V. duclouxii*-specific genes, we conducted GO and KEGG pathway enrichment analyses. The results of GO enrichment analysis indicated that *V. duclouxii*-specific genes are related to protein kinase activity, calcium-dependent phospholipid binding, ion transport, response to stress, and iron ion homeostasis. KEGG pathway enrichment analysis indicated that these genes are related to ABC transporters, sesquiterpenoid and triterpenoid biosynthesis, isoflavonoid biosynthesis, and SNARE interactions in vesicular transport (Supplementary Data Fig. S3), indicating that *V. duclouxii* may possess higher stress resistance, pH tolerance, and flavonoid content than the cultivar.

**Figure 3:**
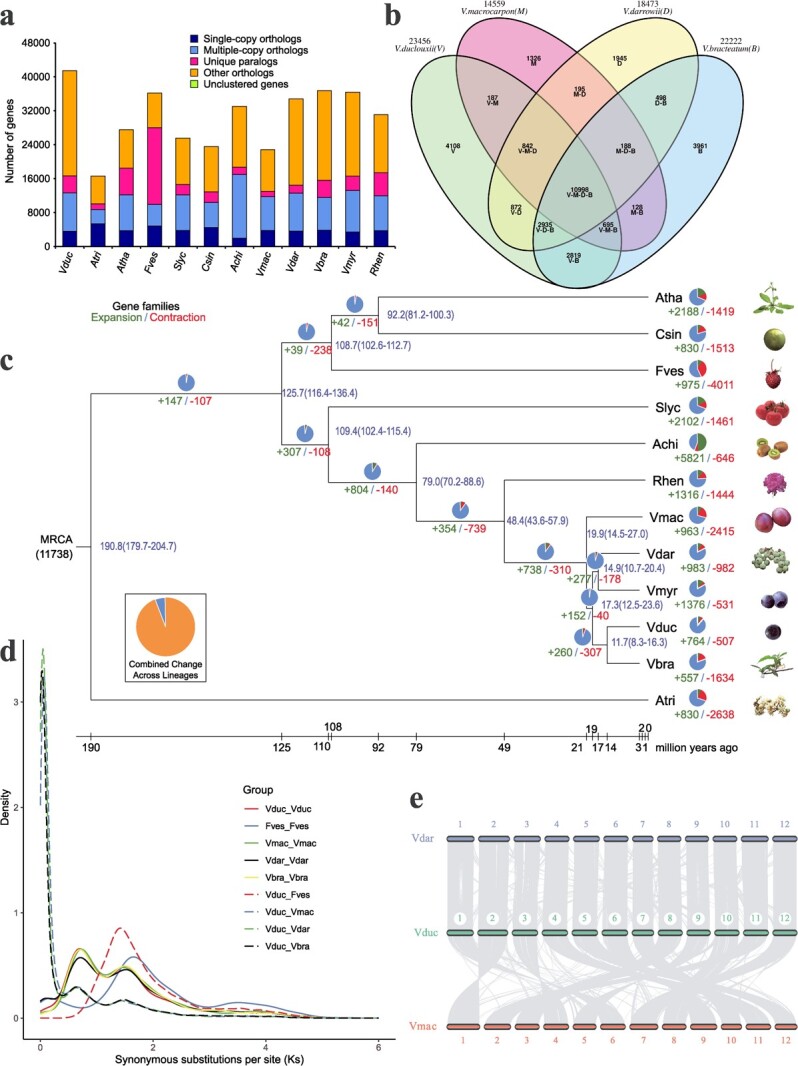
Analysis of *V. duclouxii* genome evolution. **a** Number of homologous genes in species. **b** Venn diagram showing the results of gene family clustering analysis in the four species. **c** Evolutionary tree showing the relationships among 12 species, along with divergence times. Numbers of gene families that underwent contraction (+) and expansion (-) during evolution are indicated. Atha, *A. thaliana*; Csin, *Citrus sinensis*; Fves, *Fragaria vesca*; Slyc, *Solanum lycopersicum*; Achi, *Actinidia chinensis*; Rhen, *Rhododendron henanense*; Vmac, *Vaccinium macrocarpon*; Vdar, *Vaccinium darrowii*; Vmyr, *Vaccinium myrtillus*; Vduc, *Vaccinium duclouxii*; Vbra, *Vaccinium bracteatum*. **d** Plot showing the number of synonymous substitutions per synonymous site (*K*_s_ ). **e** Diagram showing collinearity among the *V. duclouxii*, *V. darrowii*, and *V. macrocarpon* genomes.

The analysis of shared and species-specific gene families of four blueberry species (*V. duclouxii*, *V. macrocarpon*, *V. darrowii*, *V. bracteatum*) revealed that *V. duclouxii* and *V. bracteatum* possess more species-specific genes (4108 and 3961, respectively) than the other two species (Fig. 3b). The results of GO and KEGG pathway enrichment analyses of *V. duclouxii*-specific genes also showed these genes were significantly associated with ion transport, response to stress, sesquiterpenoid and triterpenoid biosynthesis, and isoflavonoid biosynthesis, further supporting its potential for higher stress resistance (Supplementary Data Fig. S4).

A rooted phylogenetic tree was constructed based on 713 single-copy genes, with *Amborella trichopoda* as an outgroup (Fig. 3c). The result indicated the *Vaccinium* and *Rhododendron* genera diverged from a common ancestor 43.6–57.9 million years ago (Mya). Subsequently, cranberry (*V. macrocarpon*) diverged from other blueberry species 14.5–27.0 Mya. *Vaccinium duclouxii* and *V. bracteatum*, which are native to China, and *V. darrowii* and *V. myrtillus*, which are mainly found in North America and Europe, diverged 10.7–20.4 Mya. *Vaccinium duclouxii* and *V. bracteatum* diverged between 8.3 and 16.3 Mya. Next, we combined the results of the phylogenetic, divergence time, and gene family clustering analyses, and predicted the contraction and expansion of gene families in *V. duclouxii* relative to its ancestors using CAFE v4.2 software. The results revealed the expansion of 764 gene families (comprising 3466 genes) and the contraction of 507 gene families (comprising 669 genes) in *V. duclouxii*.

GO and KEGG pathway enrichment analyses showed that members of the gene families that underwent expansion were related to xenobiotic detoxification by transmembrane export across the plasma membrane, defense response, protein serine/threonine/tyrosine kinase activity, glutathione transferase activity NAD + nucleosidase activity, and protein kinase activity (Supplementary Data Fig. S5), suggesting that *V. duclouxii* has higher stress resistance than other blueberry species.

The number of synonymous substitutions per synonymous site (*K*_s_), calculated for genes homologous between two species or within a single species, was used to estimate the timing of large-scale duplications. The whole-genome triplication event shared by core dicotyledonous plants occurred in *Vaccinium* species and strawberries at a *K*_s_ value of ~1.4. The *K*_s_ peak was relatively flat because of the relatively long replication time. A separate whole-genome replication event occurred in *Vaccinium* at a *K*_s_ value of ~0.6 (Fig. 3d). Next, we calculated the timing of genome-wide doubling (Mya) using the formula *T* = *K*_s_/2*r* × 10^−6^, where *r* is the nucleotide substitution rate (6.54 × 10^−9^) [28]. As a result, the doubling time was calculated as 47 Mya. Moreover, the time of occurrence of this event was very close to the time of divergence between *Vaccinium* and *Rhododendron*, suggesting that this genome duplication event potentially contributed to the differentiation of these two species. The 4-fold synonymous third codon reversal (4DTV) supports the same result (Supplementary Data Fig. S6).

Chromosome evolution in the genome of *V. duclouxii* was compared with that in the genomes of related species (*V. darrowii* and *V. macrocarpon*) using MCScan algorithms. Large-scale chromosomal rearrangements, including inversions and translocations, were detected between *V. duclouxii* and *V. macrocarpon*. Additionally, fewer scattered points were observed in the *V. duclouxii* versus *V. darrowii* comparison, suggesting a closer relationship between these two species compared with other species (Fig. 3e). Overall, these findings offer new insights into the evolution of *V. duclouxii* chromosomes.

### Soluble sugar and organic acid accumulation in *V. duclouxii* organs and fruits

**Figure 4:**
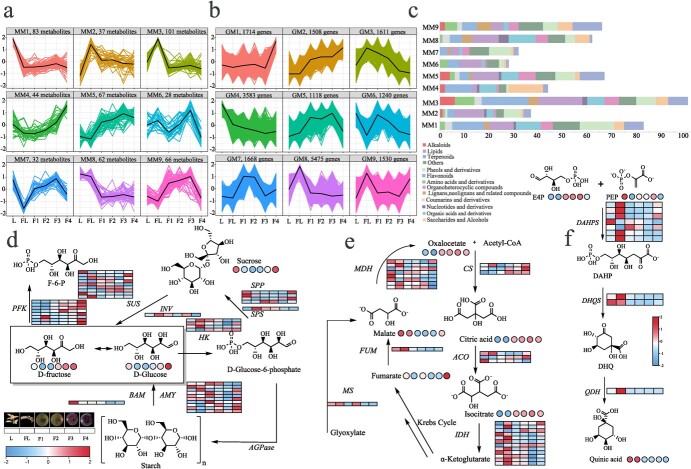
Metabolite abundance and gene expression clustering and soluble sugar and organic acid accumulation synthesis pathway. **a** Clustering of metabolites abundance based on *K*-means. **b** Clustering of gene expression based on *K*-means. **c** Statistics of the class categories of metabolites in nine MMs. **d** Soluble sugar biosynthesis pathway in *V. duclouxii*. Square boxes represent gene expression. Rounded boxes represent metabolite accumulation. Data were *Z*-score-standardized to −2 to 2. L, leaves; FL, flowers; F1, immature fruits; F2, partially ripe fruits; F3, almost ripe fruits; F4, fully mature fruits; AMY, α-amylase; BAM, β-amylase; PFK, phosphofructokinase; SUS, sucrose synthase; INV, invertase; HK, hexokinase; SPP, sucrose-6f-phosphate phosphohydrolase; SPS, sucrose-phosphate synthase; AGPase, ADP-glucose pyrophosphorylase. **e** Organic acid biosynthesis pathway (mainly citric acid and malate). MS, malate synthase; FUM, fumarase; MDH, malate dehydrogenase; CS, citrate synthase; ACO, aconitase; IDH, isocitrate dehydrogenase. **f** Quinic acid biosynthesis pathway. DAHPS, 3-deoxy-d-arabino-heptulosonate-7-phosphate synthase; DHQS, 3-dehydroquinate dehydratase; QDH, quinate dehydrogenase. The names and FPKM values of the genes in the figure are listed in Supplementary Data Table S12.

The sugar-acid ratio is one of the most important indicators influencing fruit taste, quality, and harvest time. In order to gain more insight into metabolite accumulation during *V. duclouxii* organ and fruit development and ripening, a non-targeted metabolomic analysis was conducted. A total of 520 metabolites with the Chemical Abstracts Service (CAS) registry number were identified, including 15 alkaloids, 69 flavonoids, 82 lipids, 40 sugars and alcohols, 47 terpenoids, and others (Supplementary Data Table S11). The *K*-mer clustering algorithm generated nine modules of metabolite accumulation patterns and gene expression patterns. In metabolic modules (MMs), MM7 contained metabolites with the lowest content during the flowering stage, accumulated significantly during fruit growth from the F3 stage, but decreased during the F4 stage. MM3 showed a high accumulation of metabolites during the flowering stage, but had lower content during the leaf and fruit stages. MM4 exhibited a decrease in expression during the leaf-to-flower transition, but gradually accumulated metabolites until the highest level was reached during the F4 stage (Fig. 4a). In the gene modules (GMs), GM2 showed a continuous increase in gene expression from the leaf to flower and fruit stages, while GM8 exhibited a similar expression pattern to MM3 (Fig. 4b). We also observed different distributions of metabolites in MMs. Metabolites such as terpenes were highly enriched in MM9, lipids were highly enriched in MM3, and saccharides and alcohols were highly enriched in MM4. Organic acids and derivatives were highly enriched in MM1 and MM8 (Fig. 4c).

Sweetness is one of the most important factors affecting fruit quality. Soluble sugars, including sucrose, d-fructose, and d-glucose are the main determinants of blueberry sweetness [29]. Interestingly, in *V. duclouxii*, sucrose, d-glucose, d-fructose, d-maltose, allose, d-tagatose, and d-xylose are all clustered into MM4 (Supplementary Data Table S11). A certain accumulation in leaves, with the least accumulation in the flowering period, gradually increases with fruit ripening, and undergoes a step-change accumulation during the F3 to F4 stage. This indicates that the most critical stage for soluble sugar biosynthesis is when the fruit transitions from red to black. The accumulation of soluble sugars also shows an increase in monosaccharide content, particularly a linear rise in monosaccharide content from the F3 to the F4 stage, as the fruit matures. This significant increase in monosaccharide content may increase sweetness as the fruit ripens. To further understand its biosynthetic characteristics, we analyzed the biosynthesis pathway of soluble sugars; α-amylase (AMY 6/10) and sucrose-6F-phosphate phosphohydrolase (SPP 2/4) are primarily clustered in GM1 and GM2. Their expression levels rapidly increase during the F3 to F4 stages (Fig. 4d). This indicates the pathway driving the conversion to fructose and glucose. The accumulation of soluble sugars in the later stages of fruit development is likely due to the conversion of starch into soluble sugars.

The acidity also has a great impact on fruit flavor. Research on blue bilberry and albino bilberry has shown that citric acid and quinic acid are the main organic acids in fruits [29]. In *V. duclouxii*, malate is clustered in MM8, primarily accumulating in the flowering stage, possibly due to the high energy consumption during flower opening. However, malate is less accumulated during the fruiting stage. The main biosynthesis genes, *malate synthase* (*MS*) and *fumarase* (*FUM*), are clustered in GM8, showing similar trends to malate accumulation. The expression levels of the genes involved in malate production are highly expressed during the blooming stage, and they decrease in the late fruiting stage. This could be why malate does not accumulate in large quantities in ripened fruit.

Citric acid and isocitric acid are clustered in MM5. Their content is very low during the flowering and leaf stages, but they gradually accumulate during fruit ripening, reaching the highest level during the red fruit stage, and then decrease during full ripening. The main biosynthesis gene, *citrate synthase* (*CS*), shows relatively low expression in flowers and leaves, but gradually increases during the fruiting stage. Most genes involved in the degradation pathway, such as *isocitrate dehydrogenase *(*IDH*), are clustered in GM8, with high expression during the flowering stage but gradually decreasing during the fruiting stage. This leads to citric acid and isocitric acid synthesis being greater than their degradation (Fig. 4e), which may explain the accumulation of citric acid and isocitric acid during the fruiting stage.

Quinic acid is also clustered in MM8 and accumulates significantly during the flowering and leaf stages, but its accumulation is downregulated during the fruiting stage. Its biosynthetic genes show a similar trend and mainly cluster in GM8, with high expression during the flowering and leaf stages, but decreasing expression during the fruiting stage as the fruits mature (Fig. 4f). In summary, as fruits mature, there is a gradual increase in the accumulation of soluble sugars, while organic acids decrease in accumulation as the fruit ripens, especially during the period when the fruit transitions from red to black. This trend aligns with the gradual increase in the sugar-to-acid ratio observed during blueberry ripening.

### Key regulatory factors in anthocyanin accumulation

**Figure 5:**
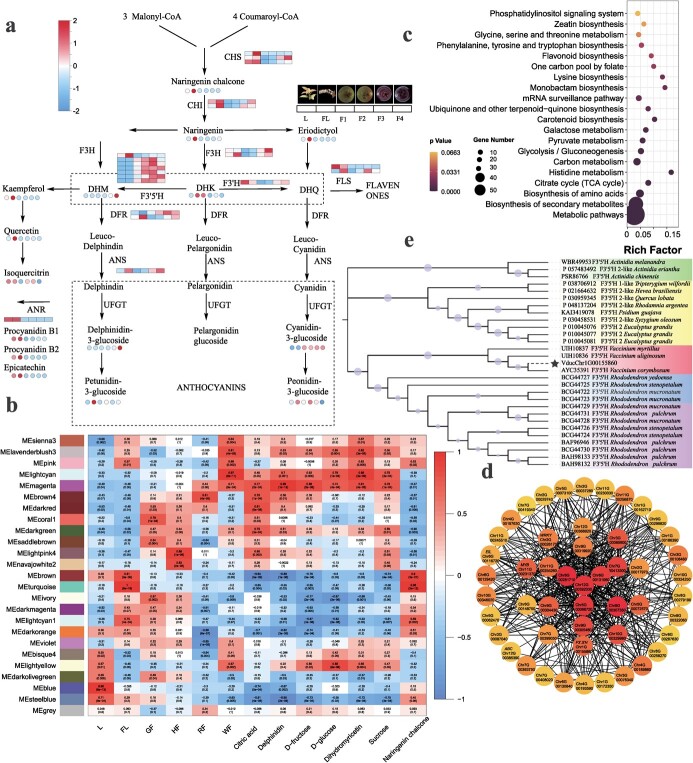
Analysis of spatial expression patterns and anthocyanin biosynthesis in *V. duclouxii*. **a** Anthocyanin biosynthesis pathway in *V. duclouxii*. Square boxes represent gene expression. Rounded boxes represent metabolite accumulation. Data were *Z*-score-standardized to −2 to 2. L, leaves; FL, flowers; F1, immature fruits; F2, partially ripe fruits; F3,almost ripe fruits; F4, fully mature fruits. CHS, chalcone synthase; CHI, chalcone isomerase; F3H, flavanone 3-hydroxylase; F3′H, flavonoid 3′-hydroxylase; F3′5′H, flavonoid-3′,5′-hydroxylase; DFR, dihydroflavonol 4-reductase; ANS, anthocyanidin synthase; UFGT, UDP flavonoid 3-O-glucosyltransferase; FLS, flavonol synthase; ANR, anthocyanidin reductase; DHM, dihydromyricetin; DHK, dihydrokaempferol; DHQ, dihydroquercetin. **b** Coexpression modules identified by WGCNA. Heat map shows the correlation between coexpression modules and various tissues/developmental stages in *V. duclouxii*. The correlation coefficient between a given module and plant sample is indicated by the text inside the cell; the upper numberrepresents the positive or negative value of the Pearson correlation coefficient (*r*) and the lower number represents the *P*-value. **c** KEGG enrichment analysis of genes in modules MEmagenta. **d** Phylogenetic tree of *V. duclouxii* and F3′5′H proteins of other species. The CDS alignment was done using MAFFT software and the maximum likelihood tree was constructed by iqtree2 with 1000 bootstrap replicates. Node numbers are bootstrap confidence values and the scale bar refers to amino acid substitutions per site. **e** Mapped networks of the top 50 hub genes constructed in the magenta module using Cytoscape software. Names and FPKM values of the genes in the figure are listed in Supplementary Data Table S12.

The most important agricultural characteristic of *Vaccinium* spp. is its high anthocyanin content. For *V. duclouxii*, the F3–F4 stage is crucial as it represents the color transition period from almost ripe to fully mature fruit, and anthocyanin biosynthesis plays a pivotal role during this period (Fig. 5a). Anthocyanins detected in *V. duclouxii* mainly existed in the form of four glycosides: delphinidin-3-glucoside, cyanidin-3-glucoside, petunidin-3-glucoside, and peonidin-3-glucoside. Additionally, metabolites like naringenin chalcone, naringenin, eriodictyol, kaempferol, quercetin, procyanidin B2, and petunidin 3-*O*-glucoside mainly cluster in MM3. They show significant accumulation in the flowering stage, increase slightly in the F3 stage but decrease in the F4 stage. Dihydromyricetin and delphinidin 3-glucoside cluster in MM4. Their content is very low in leaves and flowering stages but they gradually accumulate during fruit ripening, experiencing a rapid increase from the F3 to the F4 stage (Fig. 5a).

Consistent with the accumulation pattern of metabolites, the majority of genes involved in anthocyanin/procyanidin biosynthesis exhibit similar patterns. For instance, genes such as *chalcone synthase* (*CHS 2/3*), *chalcone isomerase* (*CHI 1/2*) and *flavanone 3-hydroxylase* (*F3H 1/1*) cluster in GM8. These genes show high expression levels during flowering. Meanwhile, at least one copy of the dihydroflavonol 4-reductase (*DFR*) and *flavonoid-3′,5′-hydroxylase* (*F3′5′H*) gene cluster in GM2, while *anthocyanidin synthase* (*ANS*) clusters in GM9, showing rapid up expression during the F3–F4 stage. This is consistent with the rapid accumulation of anthocyanins during the F3–F4 stage. Interestingly, almost all genes involved in anthocyanin biosynthesis, except for the *F3′5′H* (4/5) gene, display higher expression levels during the flowering stage, despite their flowers typically remaining colorless. This suggests that the *F3′5′H* gene and its regulation may play an important role in pigment formation in *V. duclouxii*. Copies of the *F3′5*′*H* gene, which directs the flavonoid pathway to the delphinidin branch, were primarily concentrated on Chr1, Chr6, and Chr11 in *V. duclouxii*. In addition, eight genes were identified as gene clusters on Chr1, and two genes were identified as gene clusters on Chr6 (Supplementary Data Fig. S7), which may contribute to the accumulation of a substantial amount of delphinidin in *V. duclouxii*.

To gain further insights into the relationship between metabolite accumulation and gene expression in *V. duclouxii*, we conducted a weighted gene correlation network analysis (WGCNA) using 19 448 genes (with an average FPKM >2) expressed in 18 samples. A threshold of 12, which provided the best fit for the scale-free topological index, was selected. A total of 25 coexpression modules were identified based on similar expression patterns. The heat map of module–trait correlations reveals a strong positive correlation (*R* = 0.99) between the expression of genes in the magenta module and the accumulation of delphinidin in metabolites (Fig. 5b). Interestingly, the accumulation of soluble sugars, such as sucrose, fructose, and glucose, and organic acids like citric acid showed a high correlation with the magenta module. This suggests a similar mechanism underlying the accumulation of delphinidin and soluble sugars. Further KEGG analysis of genes within the magenta module confirmed these findings. Enriched pathways within the magenta module included galactose metabolism, pyruvate metabolism, glycolysis/gluconeogenesis, and the citrate cycle (TCA cycle), all of which are associated with soluble sugar synthesis and metabolism. Additionally, pathways related to flavonoid synthesis, such as flavonoid biosynthesis, and carotenoid biosynthesis were also enriched (Fig. 5c).

The Cytohubba plugin in Cytoscape was employed to identify hub genes and visualize the top 50 genes with high connectivity in the MEmagenta module (Fig. 5d). Remarkably, our analysis revealed a significantly connected putative *F3′5′H* gene (*VducChr1G00155860*), which exhibited high connectivity within the hub gene network and held a central position within the hub gene (Fig. 5d). Phylogenetic analysis demonstrated that *VducChr1G00155860* shares high homology with the *F3*′*5′H* gene (*AYC35391*) in *V. corymbosum*, the *F3′5′H* gene (*KAH7844363*) in *V. darrowii*, and the *F3′5′H* gene (*UIH10837*) in *V. myrtillus* (Fig. 5e). These findings suggest that this gene may potentially serve a similar function in *V. duclouxii.* Additionally, among the top 50 highly connected genes, we identified *MYB* (*VducChr5G00073100*), *WRKY* (*VducChr2G00026170*), and *EIL* (*VducChr6G00116720*). *VducChr5G00073100* exhibits homology with *AtMYB21* in *A. thaliana*. In *Arabidopsis*, AtMYB21 interacts with the key transcription factor MYC2 in the jasmonic acid pathway and plays an important role in jasmonic acid-mediated secondary metabolite synthesis [30, 31]. Furthermore, *EIL* gene plays a critical role in ethylene signal transduction.

We further conducted promoter analysis on highly expressed key genes involved in anthocyanin biosynthesis and found a large number of response hormone elements in their promoters, such as abscisic acid (ABA) responsiveness, auxin-responsive element, gibberellin-responsive element, MeJA-responsiveness, and other response elements. This suggests that hormones may play an important role in regulating and synthesizing anthocyanins. Further analysis revealed the presence of numerous Myb/SANT/MYB binding sites on their promoters. This suggests that the *MYB* gene may play an important role in anthocyanin synthesis (Supplementary Data Fig. S8). To further validate the crucial role of transcription factors in anthocyanin biosynthesis, we identified the *MYB* gene family, which plays a vital role in anthocyanin biosynthesis. In the whole genome, we identified 280 *MYB* genes, and the expression of four of these *MYB* genes clustered in the MEmagenta module in WGCNA. Among these four genes, two (*VducChr1G00175800* and *VducChr8G00267910*) are homologous to *AtGLK1* and *AtMYB114* in *Arabidopsis*, the AtGLK1 promotes anthocyanin biosynthesis through an MBW complex-dependent pathway [32]. AtMYB114 regulates the expression of later steps in anthocyanin biosynthesis [33].

## Discussion

*Vaccinium duclouxii*, an exclusively Chinese species, is extensively found in Yunnan Province. Its fruit’s nutritional components surpass those of rabbiteye blueberries, rendering it a valuable resource for both dietary and medicinal purposes [10]. However, the limited research and the absence of a complete genome pose significant constraints on its potential applications.

In this study, we present the first gap-free genome assembly of *V. duclouxii*, achieved by integrating long-read and Hi-C sequencing technologies. Our assembly exhibits a remarkable overlap group N50 of 48.51 Mb, surpassing all other available genome assemblies of *Vaccinium* species [13–17]. Among the 12 chromosomes of *V. duclouxii*, all chromosomes were entirely gap-free. Such an assembly provides unique value for further genomic and functional studies and molecular breeding.

Although advanced metabolite detection techniques have identified numerous compounds associated with fruit quality, the precise regulatory networks controlling these compounds remain unclear. Supported by the high-quality genome of *V. duclouxii*, combined transcriptomic and metabolomic analyses revealed a significant accumulation of soluble sugars, especially sucrose, fructose, and glucose, during the transition from almost ripe to fully mature stages. This is similar to the sugar accumulation process during the development and maturation of *V. myrtillus* berries [34]. This indicates that the transition period from almost ripe to fully mature is critical for soluble sugar synthesis, with the highly expressed AMY and SPP enzymes likely playing crucial roles in the accumulation of fructose and glucose. Similar results were observed in *V. corymbosum* [35]. Organic acids such as citric acid and malate determine the fruit’s acidity; moderate acidity can enhance the flavor of the fruit, while excessive acidity tends to reduce fruit quality. Studies on *V. corymbosum* have shown that citric acid, malate, and quinic acid decrease as blueberries ripen [35]. Similarly, *V. duclouxii* exhibits a declining trend in malate and quinic acid accumulation during fruit ripening. However, citric acid and isocitrate accumulate significantly throughout fruit maturation, reaching their peak levels at the almost ripe stage and slightly decreasing thereafter. These findings may shed light on why *V. duclouxii* maintains a relatively high level of total acidity and possesses unique flavor during full ripening.

*Vaccinium duclouxii* flowers are primarily white, occasionally pink, and the berries transition from green to semi-green, red, and finally purplish-black during ripening. This change in color is accompanied by the biosynthesis and accumulation of anthocyanins, typically due to the upregulation of genes that encode core anthocyanin biosynthesis enzymes [36]. These enzymes include PAL, CHI, F3H, F3′H, F3′5′H, DFR, and ANS. Our study identified multiple copies of *F3′5′H* genes, which cluster on chromosomes, potentially contributing to the high delphinidin content in *V. duclouxii*. During the flowering stage, the expression of *F3′5′H* was minimal, while genes involved in flavonol and proanthocyanidin production exhibited high expression levels, resulting in predominantly white-colored flowers.

Our research has found that during the flowering period, many genes involved in anthocyanin synthesis (excluding *F3′5′H*) exhibit higher expression levels compared with fruit ripening. The main function of the F3′5′H protein is to direct the biosynthesis of flavonoids toward the delphinidin branch and its derivatives. Delphinidin and its derivatives are the main anthocyanins found in most *Vaccinium* species. Delphinidin-3-*O*-galactoside, for instance, is the primary anthocyanin found in *V. corymbosum* [37]. These compounds serve as precursors for producing blue and purple pigments [38]. This suggests that *F3′5′H* may be a key gene in *Vaccinium* species. During the fruiting period, especially during the F3–F4 stages, the high expression of the *F3′5′H* gene promotes fruit coloring, similar to the accumulation of anthocyanins observed in *V. corymbosum* during the maturation stages [39].

Moreover, by using the WGCNA package to analyze the correlation between genes and metabolites, we identified a highly interconnected *F3′5′H* gene in the magenta module. Evolutionary analysis shows this gene clusters with *F3′5′H* genes from other *Vaccinium* species, indicating its crucial role in the biosynthesis of delphinidin and its derivatives. During the ripening process of *V. myrtillus* berries, the upregulation of F3′5′H expression suggests a shift towards the flavone pathway in the biosynthesis of delphinidin [40]. In the cultivated blueberry variety ‘Misty’, the *F5′3′H* genes have been recognized as crucial genes involved in the synthesis of flavonoids [41]. Intriguingly, the magenta module also exhibits a strong correlation with soluble sugars, particularly fructose. Previous studies have indicated that sugars (such as sucrose, fructose, and glucose) can act as signaling molecules and major hormonal-like signals for anthocyanin synthesis [42]. For instance, in the pear variety ‘Hongtaiyang’ anthocyanins display a significantly positive correlation with fructose [43]. Similarly, sugars may play a role in signaling the synthesis of anthocyanins in apricots [44]. Sugars not only provide carbon sources and structural components for anthocyanin biosynthesis but also enhance the expression levels of structural genes and regulate *MYB* genes [43, 45].

Among the top 50 hub genes, we also identified a homolog of *ArabidopsisMYB21*, which is involved in the jasmonic acid pathway, as well as *EIL*, which participates in ethylene metabolism. Studies have shown that applying ABA, ethylene, and JA externally can enhance the accumulation of anthocyanins in apples [46], suggesting that these genes may be closely related to the accumulation of delphinidin and its derivatives.

Furthermore, within the magenta module, we have identified and annotated 24 transcription factors, including two bHLHs (VducChr12G00384050; VducChr6G00116900) and four MYBs. Two of the MYBs are considered homologs capable of forming the R2R3-MYB/bHLH/WD40 (MBW) complex. We also observed the enrichment of three *WD40* genes (*VducChr2G00038400*, *VducChr1G00186370*, *VducChr9G00297710*) in the MEmagenta module. MYB, bHLH proteins, and WD40 proteins, acting as scaffolds, form the MBW complex, which regulates flavonoid synthesis [47, 48]. Interestingly, in monocotyledonous plants, MYB collaboratively regulates early flavonoid biosynthetic genes, while in dicotyledonous plants MYB directly regulates early biosynthesis genes referred to as EBGs (e.g. *CHS*, *CHI*, *F3H*, and *F3′H*), and the MBW complex primarily controls late biosynthesis genes referred to as LBGs (e.g. *DFR*, *ANR*, *UFGT*), thus regulating downstream accumulation of anthocyanins through the MBW complex [49]. The coordinated regulation of sugars and hormones by the R2R3-MYB/bHLH/WD40 (MBW) complex plays a crucial role in the regulation of anthocyanin biosynthesis pathways in *Arabidopsis* [42]. The binding of this MBW complex is likely associated with the regulation of F3′5′H.

Moreover, *V. duclouxii* typically flourishes in high-altitude environments exposed to heightened ultraviolet (UV) radiation. The conspicuous elevation of the total sugar content ratio and the accumulation of anthocyanins in *V. duclouxii* are presumably associated with their adaptability. Soluble sugars play an indispensable role not only in the direct antioxidant defense mechanisms of plants [50], but also in synergistically amplifying plant resilience against UV radiation when combined with other safeguarding compounds like flavonoids. Investigation into the *Vaccinium uliginosum* population suggests that populations thriving at loftier altitudes generally manifest escalated levels of fructose, glucose, and overall sugars [51]. Additionally, blueberries subjected to measured UV-B dosages have a tendency to amass heightened levels of sugars and anthocyanins [52]. Furthermore, heightened anthocyanin content contributes to UV protection. Downstream genes participating in the pathway of anthocyanin synthesis in blueberries exhibit responsiveness to UV-B exposure, prompting heightened anthocyanin accumulation [53]. This UV-B treatment stimulates the heightened expression of blueberry genes *VcMYBA2* and *VcMYB114*, subsequently leading to an elevation in anthocyanin glycoside content [54]. Nevertheless, certain studies advocate that the accumulation of anthocyanins is predominantly under the sway of developmental and genotypic factors, with altitude-associated environmental influences exerting minimal regulatory impacts [55]. Consequently, delving deeper into the mechanisms governing the conspicuous sugar-to-acid ratio and anthocyanin buildup in *V. duclouxii* will contribute to a more holistic understanding of their adaptive traits.

In summary, completing the T2T genome of *V. duclouxii* can help us further explore its excellent traits, including high sugar and acidity levels, as well as the regulatory mechanisms behind its high anthocyanin content. These characteristics are of significant importance in blueberry breeding and production. Additionally, this research enables the discovery of excellent genes in *V. duclouxii* related to stress resistance. It is expected that this study will have a significant impact on future blueberry breeding and production.

### Conclusions

Here, we report the first gapless T2T genome assembly of *V. duclouxii*, along with detailed genomic information, which provides a foundation for breeding new high-quality blueberry varieties. This study not only has strong scientific research significance and good industrial value, but also lays the foundation for improving and cultivating high-quality blueberry varieties in the future.

## Materials and methods

### Plant materials and sequencing

This study used *Vaccinium duclouxii* trees grown in the germplasm resource nursery at the High Mountain Economic Plant Research Institute in Lijiang City, Yunnan Province, China. Fresh, healthy, young leaves were harvested from 2-week-old branches, and mature leaves, flowers, and fruits at different ripening stages (immature fruits, partially ripe fruits, almost rape fruits, and fully mature fruits) were harvested. Immediately after collection, the samples were placed in a freezing chamber with liquid nitrogen and stored at −80°C for future use. Three biological replicates were used for subsequent transcriptome analysis and five biological replicates were used for metabolome profiling.

High molecular weight genomic DNAs (gDNAs) were extracted from young leaves using the CTAB method, and evaluated using a NanoDrop One spectrophotometer (NanoDrop Technologies, Wilmington, DE, USA) and Qubit 3.0 Fluorometer (Life Technologies, Carlsbad, CA, USA). Each SMRTbell library was prepared from 50 μg of gDNA using the SMRTbell Express Template Prep Kit 1.0. PacBio HiFi sequencing was performed on the PacBio Sequel II platform (Pacific Biosciences, CA, USA) according to the manufacturer’s instructions. For ONT ultra-long sequencing, the library was prepared using the Oxford Nanopore SQK-LSK109 kit, and then sequenced on a PromethION flow cell (Oxford Nanopore Technologies, Oxford, UK). The Hi-C sequencing libraries were prepared and sequenced as described previously [56]. Sequencing was conducted by a commercial company (Benagen Technology, Wuhan, China).

### Genome assembly and assessment

The ONT ultra-long sequence reads were assembled with NextDenovo (v2.5.0) (https://github.com/Nextomics/NextDenovo) software using the following parameters: read_cutoff = 1 k; xtgraph_options = 1 g; xtgraph_options = −a 1. Two assembly strategies were employed for the HiFi data, including pure HiFi assembly and mixed HiFi + ONT ultra-long [57]. Minimap2 v2.17 was used to align the mitochondrial and chloroplast sequences and remove sequences with >50% base-pair alignment [58]. Bacterial contamination was eliminated by utilizing the BLAST refseq database, and contigs with low read support were also removed.

To identify the association between different contigs, Hi-C interaction relationships were used, and the contigs were clustered. The genome sketch with a 2*n* karyotype was generated using ALLHiC v0.9.8 software through agglomerative hierarchical clustering [59]. The interaction between contigs was transformed into a specified binary file using 3D-DNA (v180419) [60] and juicer (v1.6) [61]. Juicebox v1.11.08 was used for visualizing and guiding manual sequencing and orientation of contigs [62]. The gap regions in the genome were filled by utilizing winnowmap (v1.11, parameters: k = 15, –MD) to align the gap-filling data [63], including ONT ultra-long raw sequencing data, HiFi raw sequencing data, nextDenovo, Necat, and HIFI assembly data, with the gap regions in the genome. If the alignment could span across both ends of the gap, the longest and most optimal alignment region was selected for replacement. Subsequently, the patched version of the genome containing the filled gaps was aligned with HiFi reads with a size ≥10 kbp using Winnowmap2 (parameters: k = 15, greater-than distinct = 0.9998, -MD, −ax map-pb) [64]. The alignment information was filtered using other software tools, and finally error correction was performed using racon (v1.6.0, options: -L -u, available at https://github.com/isovic/racon/tree/liftover). HiCExplorer software (v3.6) was utilized to draw the relationship between contig interaction strength and position [65].

### Identification of telomeres and centromeres

In most plants, telomere sequences consist of small conserved satellites arranged in tandem. A plant telomere sequence (AAACCCT) was identified, and a telomere pipeline was developed using VGP (https://github.com/VGP/vgp-assembly ) to identify telomeres on all 12 chromosomes. The telomere sequences (HiFi and ONT reads) were manually identified and repaired. The *V. duclouxii* assembly was polished using both Racon and Merfin, with six iterative rounds [66]. Centromeres were detected using Centromics software (https://github.com/ShuaiNIEgithub/Centromics) and the quarTeT webtool [67], which identifies high- and low-density genes with short tandem repeats, typical features of the centromeric region. These features were used to identify continuous clusters with candidate centromeric tandem repeats.

### Genome annotation

To identify and classify repetitive sequences in the genome, RepeatModeler v1.0.11 was used to identify and classify repetitive sequences [68]. The *de novo* and known duplicate libraries were merged, and RepeatMask v4.0.9 was used to predict repetitive sequences and TE types throughout the genome [69]. All predicted repeat sequences were integrated.

BUSCO [70] was used to assess the *V. duclouxii* genome integrity, and genomic continuity was evaluated by calculating the N50 length of contigs. The accuracy of the genome assembly was evaluated by mapping whole-genome shotgun (WGS) sequencing data to the genome using BWA-MEM [71] and calculating the mapping rate and coverage using qualimap2 [72]. The LAI values were evaluated for the assembled genome using repetitive sequences [73].

Gene structure was predicted using a combination of homology-based prediction, *de novo* prediction, and transcriptome-based prediction. Exorate v2.4.0 was used to perform homology-based prediction, with the selected protein sequence set of related species [74]. *De novo* prediction was performed using Augustus v3.3.2 [75] and GlimmerHMM v3.0.4 [76], based on the training set obtained with BUSCO v5.2.2 [77]. Next, transcriptome evidence included *de novo* assembly of transcripts based on Trinity v2.6.6 [78] and genome-guided assembly by HISAT2 v2.1.0 [79] and StringTie v2.1.4 [80]. Then, open reading frames (ORFs) were predicted using TransDecoder v5.1.0, and a complete and non-redundant gene set was obtained by integration and deredundancy.

Motifs, domains, protein functions, and metabolic pathways were predicted using existing databases such as KEGG, GO, and UniProt. Additionally, tRNAs were predicted using tRNAscan-SE v1.23 [81]; rRNA prediction was performed using rRNA databases; and ncRNAs were searched using INFERNAL v1.1.2, based on the Rfam database [82].

### Analysis of genome evolution

To investigate the relationship between biological evolution and specific biological functions in *V. duclouxii*, gene family clustering analysis was carried out using OrthoFinder v2.3.12 [83], followed by functional annotation of the identified species-specific gene families using the R package clusterProfiler for GO and KEGG annotations [84]. A maximum likelihood (ML) phylogenetic tree, based on supergenes, was constructed using RaxML software (v8.2.10) [85]. Gene family contraction and expansion analysis were conducted using CAFÉ v3.1, based on the results of evolutionary tree and gene family clustering analyses [86].

The *K*_s_ method was used to identify genome-wide replication events, where the synonymous mutation frequency (*K*_s_), non-synonymous mutation frequency (*K*_a_), and *K*_a_/*K*_s_ ratio of a collinear gene pair was calculated using the yn00 module in PAML v4.9 [87]. A gene density map was drawn using ggplot2 (v2.2.1) [88] for species closely related to *V. duclouxii*, and JCVI (v0.9.13) was used to draw a collinearity diagram of the current patterns of these species [89].

Positive selection analysis was performed using the CodeML subroutine in PAML v4.9 [87], based on the branch-site model, followed by likelihood ratio tests (LRTs) on two models (model A and null model) using the chi2 program in PAML to obtain significant differences (*P* < .05). Genes that were significantly positively selected were identified. Finally, collinear blocks were identified using MCScanX (parameters: - a - e 1e-5 - s 5) [90], and a circular diagram was drawn using the R package circle [91].

### Metabolome, transcriptome, and gene family analysis

Metabolome profiles were produced using a non-targeted metabolomics method. Samples were freeze-dried and ground into fine powder, then the powder was mixed with 1.0 mL of 70% aqueous methanol and kept at 4°C overnight. The mixture was subsequently centrifuged at 10 000 g for 10 minutes. After centrifugation, the supernatant was collected, filtered and subjected to LC–MS/MS analysis (Q Exactive Orbitrap, Thermo Fisher Scientific, USA).

RNA-seq data were analyzed using HISAT2 v2.1.0 [79] and Stringtie v2.1.4 [80]. ORFs were predicted using TransDecoder software (v5.1.0). Differential gene expression between sample groups was performed using DESeq2 v1.10, with an identification threshold of |log2FoldChange| > 1 and *P*_adj_ <.05 [92]. The GO and KEGG enrichment analyses of differentially expressed genes (DEGs) were performed using clusterProfiler v4.0 [84].

To identify modules with high gene correlation, co-expression network analysis was performed using the WGCNA v1.703 package of R v3.6.3 [93]. Transcripts with an average FPKM value of <2 were filtered out, and modules related to phenotypic traits in the reconstructed network were identified by converting the adjacency matrix into a topological overlap matrix. The potential regulatory sites on the promoter were analyzed using plantCARE (http://bioinformatics.psb.ugent.be/webtools/plantcare/html/); furthermore, the Myb/SANT/MYB binding sites were analyzed using the online platform PlantPAN3.0 (http://plantpan.itps.ncku.edu.tw/index.html). The screening of hub genes was performed using the CytoHubba plugin [94], and visual analysis was conducted using Cytoscape v3.8.2 [95].

HmmerSearch 3.0 software was used to obtain all gene families [96], and MAFFT v7.158 was used to compare the amino acid sequences using the —auto parameter [97]. ML methods were used to ensure the accuracy of the phylogenetic tree, and an ML phylogenetic tree was constructed using iqtree2 with a self-expanding value of 1000 [98]. CDD was used to predict conserved domains (https://www.ncbi.nlm.nih.gov/cdd/), and tbtools [99] and online tools (https://www.chiplot.online/) were used to visualize the results.

## Supplementary Material

Web_Material_uhad209
