# Supplementary material for: The telomere-to-telomere gap-free reference genome of wild blueberry (Vaccinium duclouxii) provides its high soluble sugar and anthocyanin accumulation
Source: Hortic Res. 2023 Oct 10;10(11):uhad209. doi: 10.1093/hr/uhad209 (PMC10681038; doi:10.1093/hr/uhad209)
Supplement: Web_Material_uhad209 [file web_material_uhad209.zip › Supplementary_Figures109.docx]

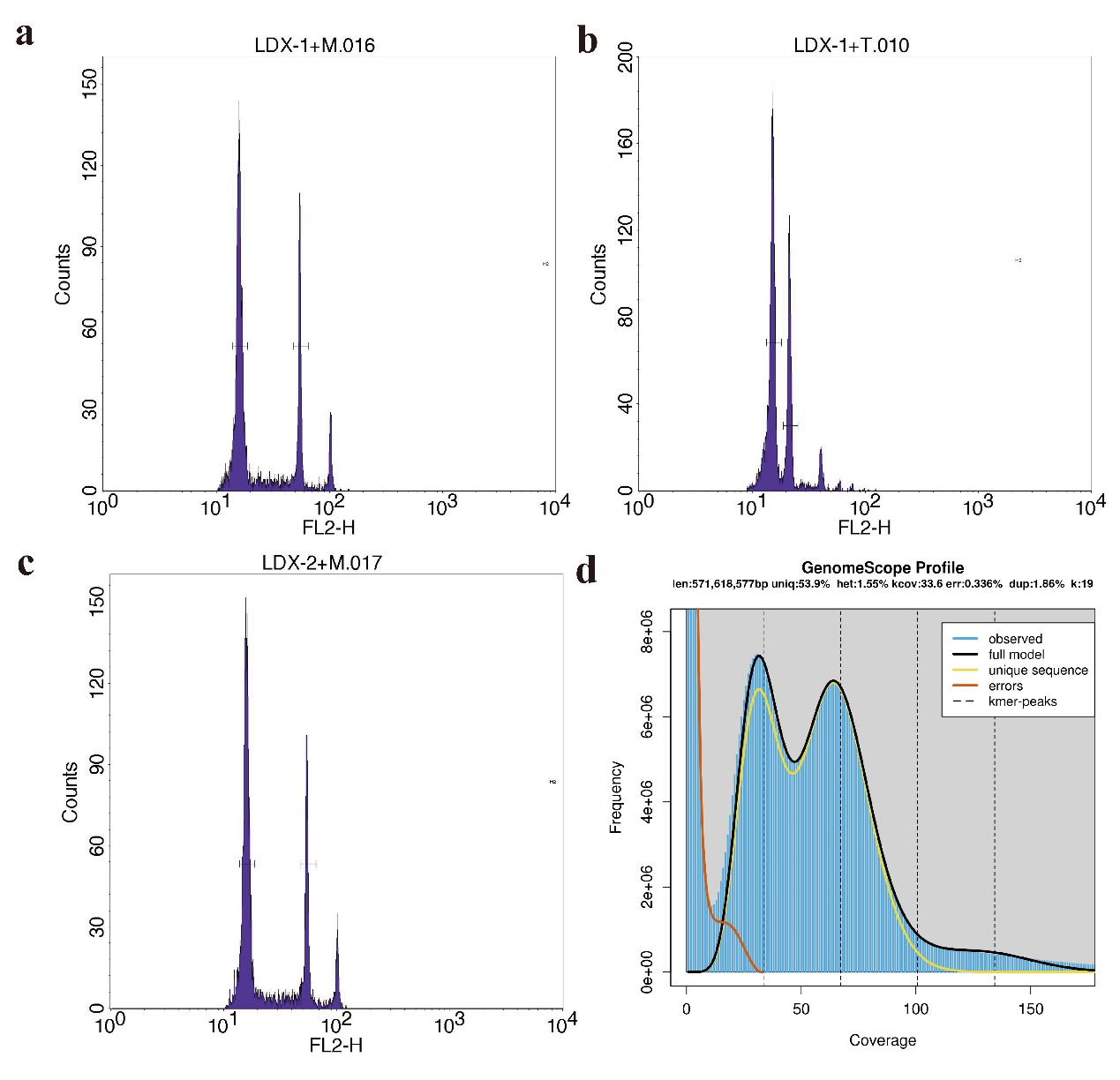


**Figure S1 Flow cytometry and distribution map for genome size determination**

**a-c** Flow cytometry was used to determine genome size, tomato, maize (B73) reference genome was selected as the internal reference **b** Kmer distribution map. Genome survey indicated the genome of *V. duclouxii* was about 571.62 Mb with 1.55% heterozygosity


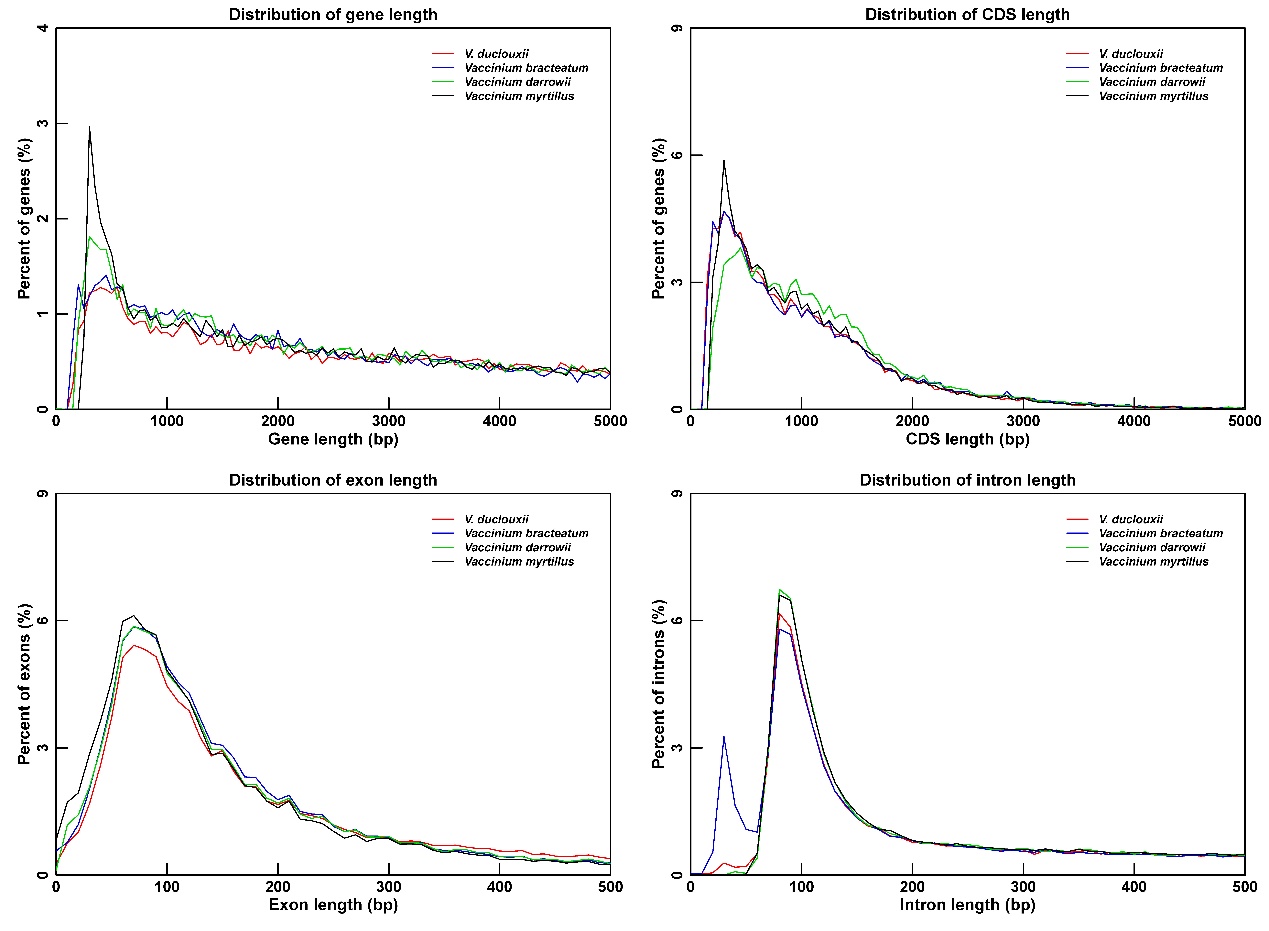


**Figure S2 Comparison of genes in closely related species**


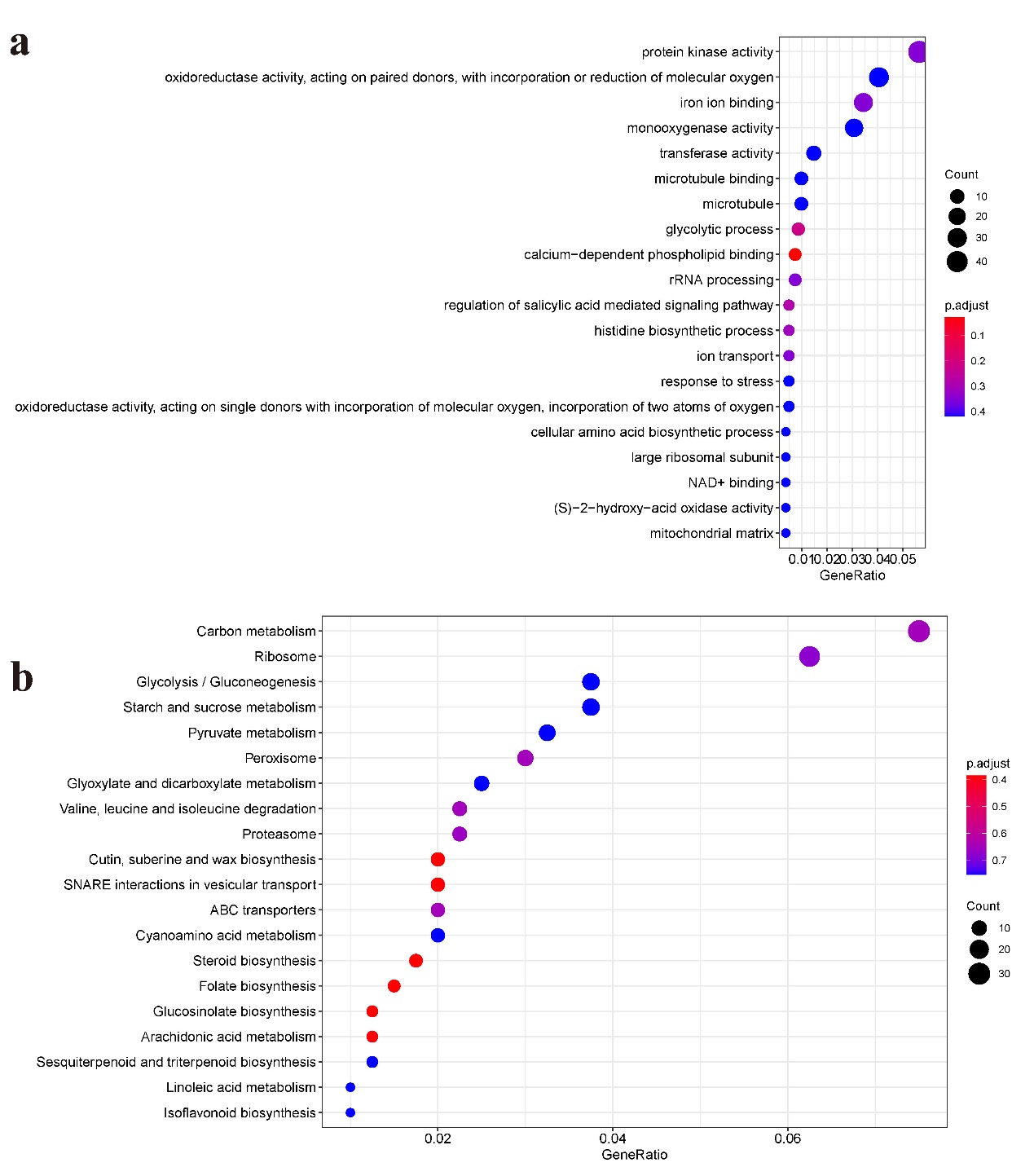


**Figure S3 GO and KEGG enrichment for unique gene families of the genus *Vaccinium***


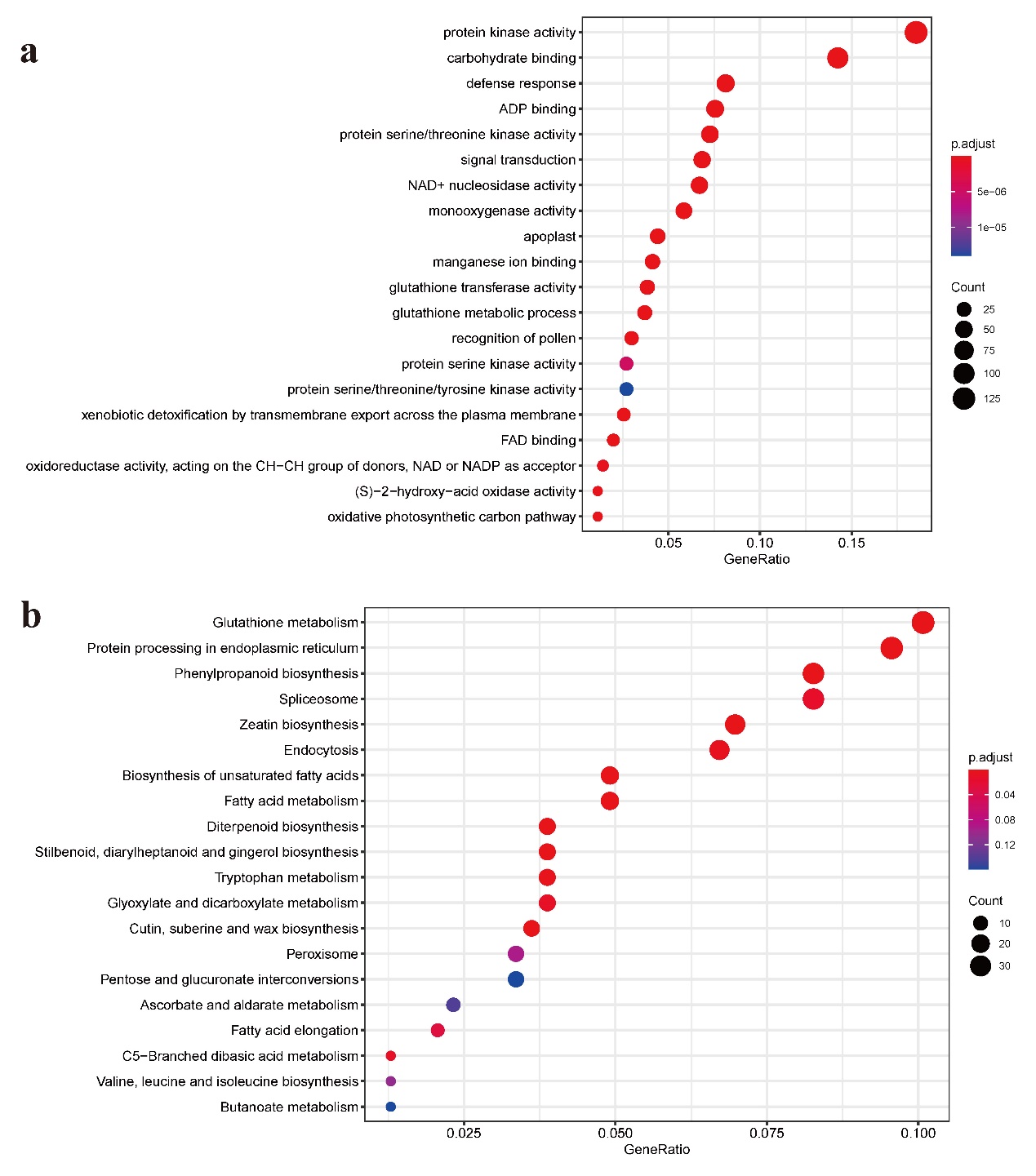


**Figure S4 KEGG and GO enrichment analysis of expanded genes.**

a KEGG enrichment analysis of contracted genes b GO enrichment analysis of expanded genes


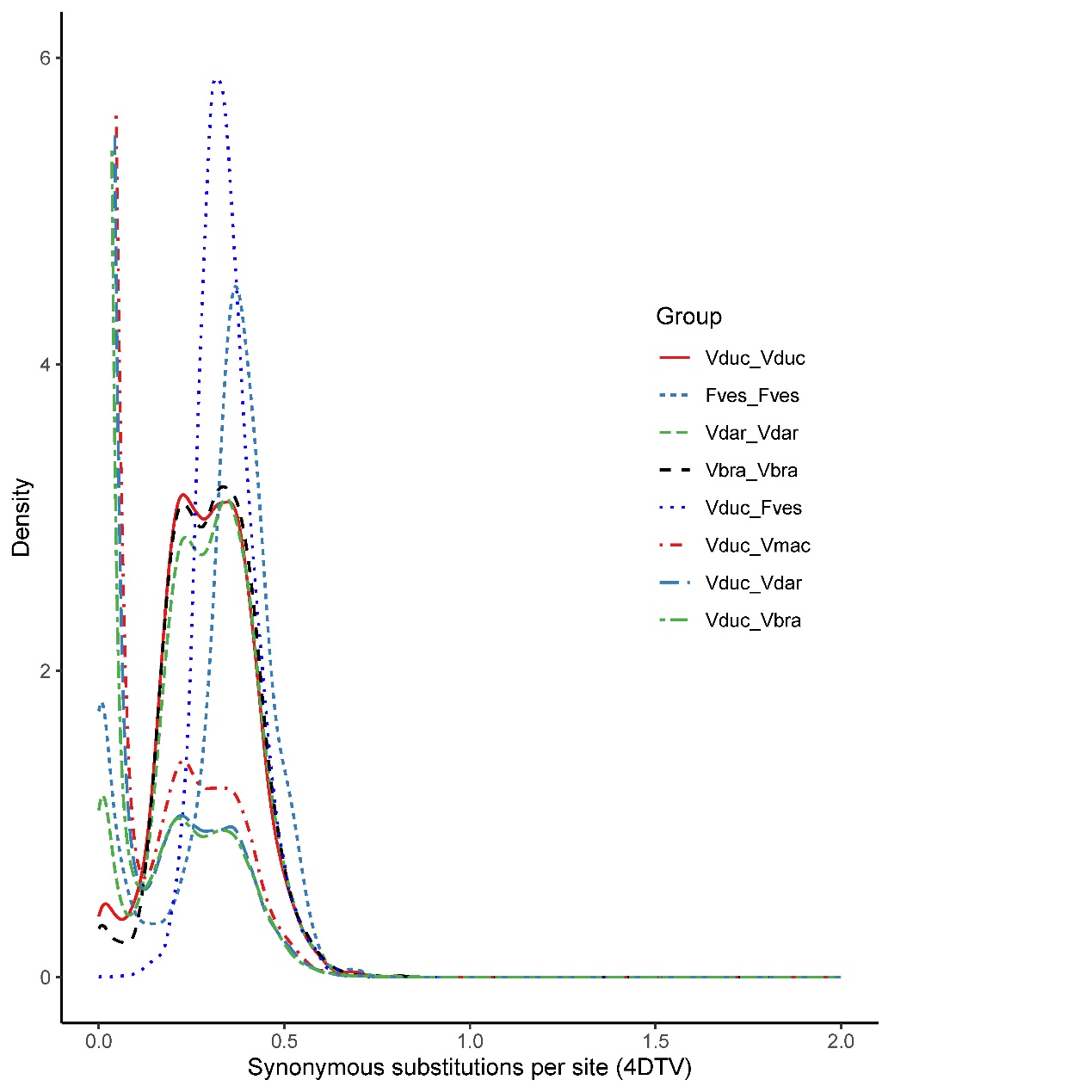


**Fig S5 4DTV plot of WGD events detection of species and *V. duclouxii***


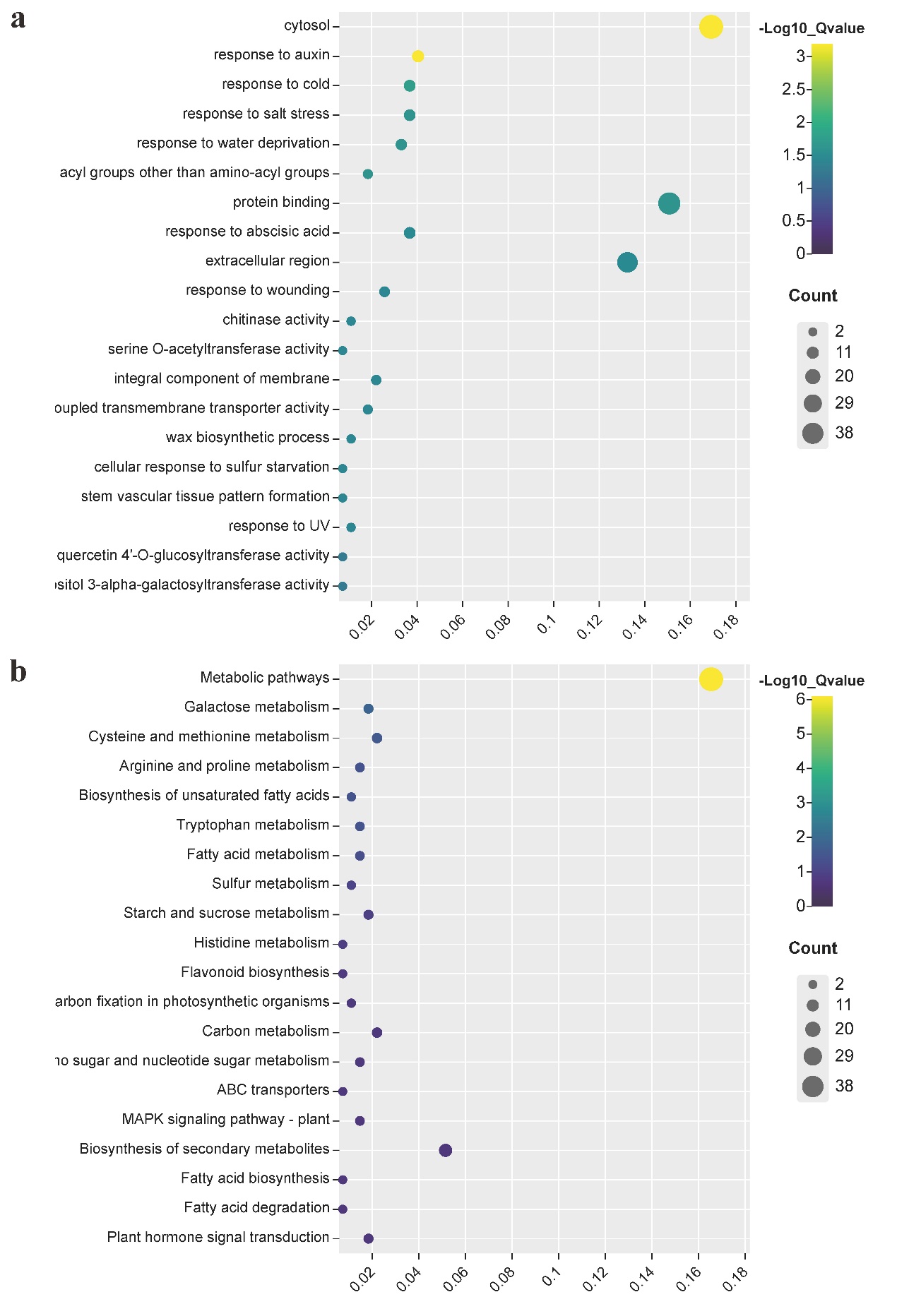


**Figure S6 WGCNA modules gene functional enrichment analysis**

**
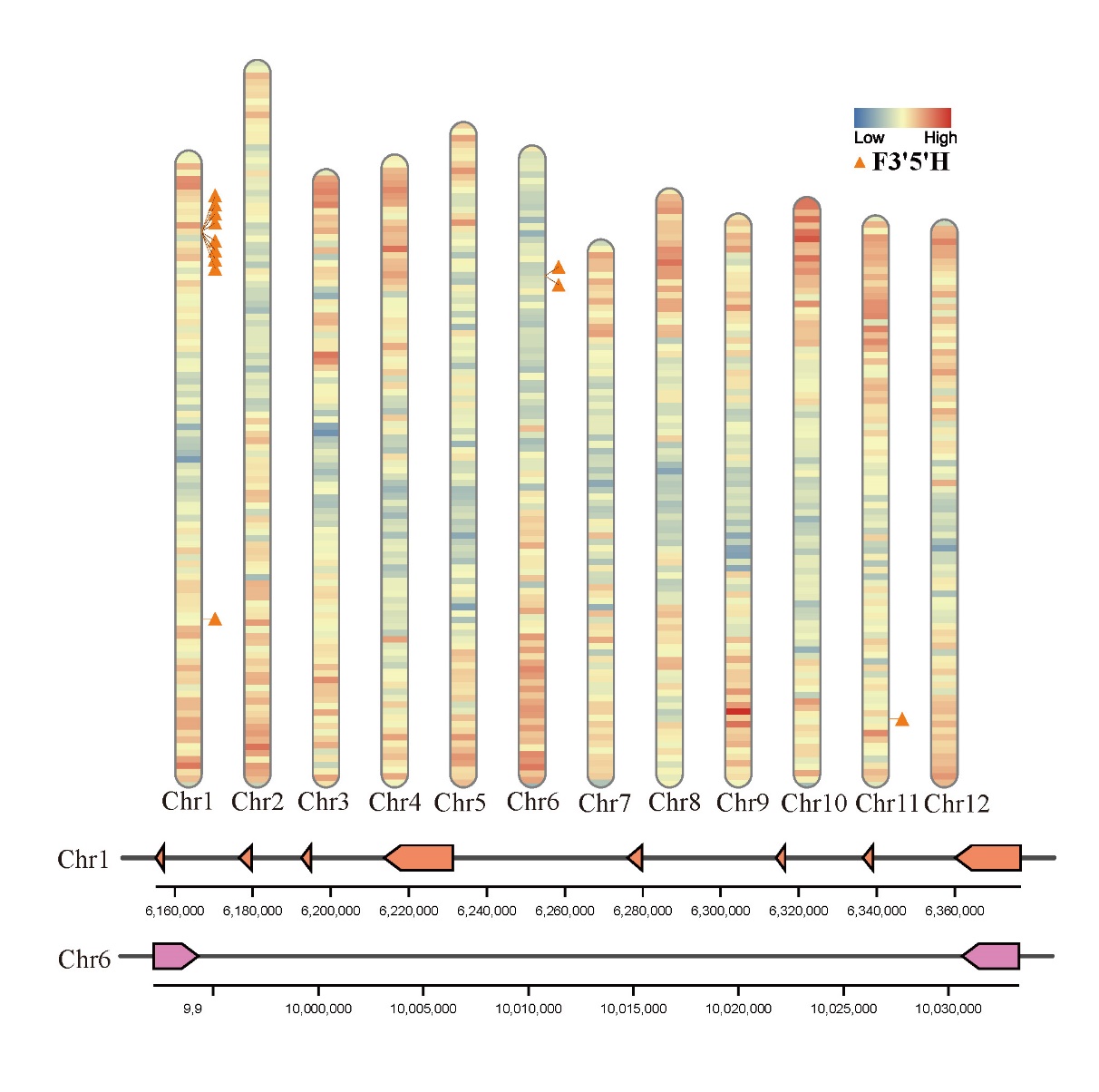
**

**Figure S7 Chromosomal distribution of F3'5'H genes.**

Eight F3'5'H genes are clustered on chr1. Two F3'5'H genes are clustered on chr6

**
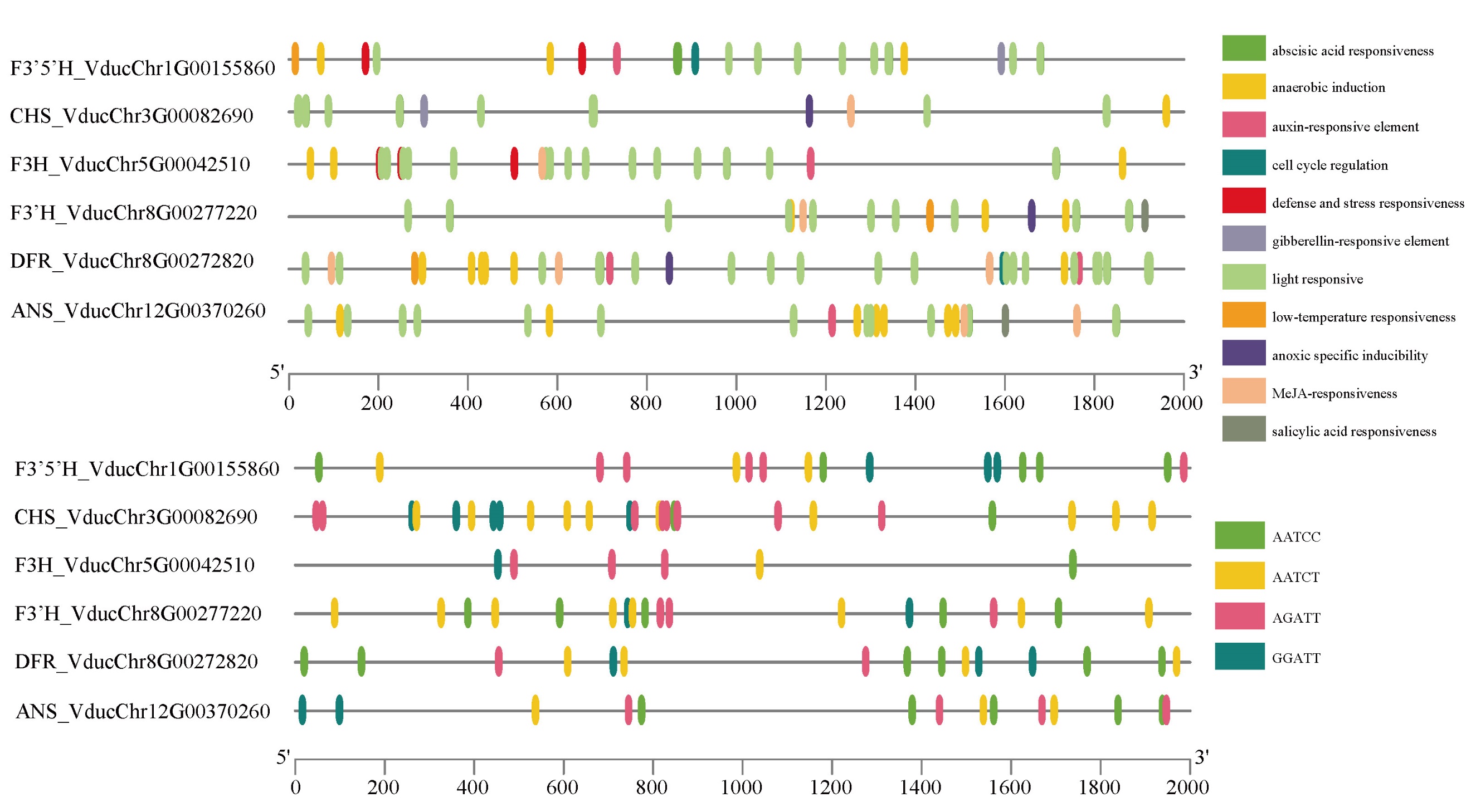
**

**Figure S8 Analysis of the cis-regulatory promoter elements in promoters of the key anthocyanin biosynthesis genes.**

The associated cis-elements and their known biological functions were performed using the PlantCARE database. The cis-regulatory elements Myb/SANT; MYB were performed using web PlantPAN3.0.
